# Mercury poisoning caused by Chinese folk prescription (CFP): A case report and analysis of both CFP and quackery

**DOI:** 10.1097/MD.0000000000005162

**Published:** 2016-11-04

**Authors:** Tingting Mo, Si Sun, Yongyi Wang, Dong Luo, Bin Peng, Yinyin Xia

**Affiliations:** a Department of Occupational and Environmental Hygiene, School of Public Health and Management, Research Center for Medicine and Social Development, Innovation Center for Social Risk Governance in Health, Canada–China–New Zealand Joint Laboratory of Maternal and Fetal Medicine, Chongqing Medical University; b Department of Occupational Medicine, Chongqing Prevention and Treatment Center for Occupational Diseases; c Department of Statistics, School of Public Health and Management, Chongqing Medical University, Chongqing, China.

**Keywords:** case report, Chinese folk prescription, mercury poisoning, oral, traditional Chinese medicine

## Abstract

**Background:**

Though as a heavy metal, mercury has a long history in the culture of traditional Chinese medicine (TCM). Also until now, we can still find evidence of mercury in some Chinese folk prescriptions (CFP)s in China.

**Case:**

We report a case of a 35-year-old rural woman, who took almost 35 g of Hg_2_O orally to treat her itchy skin followed a folk prescription of an unlicensed practitioners engaging in quackery (UPEQ), which lead to dark red bloody stool and mucus, nausea, and numbness. She sought help from some general hospitals, which brought her only misdiagnosis. Only after a mercury level test conducted by Chongqing Prevention and Treatment Center for Occupational Diseases (CPOD) confirmed her mercury intoxication, she was treated with chelation therapy with sodium dimercaptosulphonat and showed significant improvements.

**Conclusion:**

Confused by differences between TCM and CFP, people might take dangerous remedies without realizing the consequences; not only could it exacerbate their primary disease, but it could lead to unexpected and disastrous results.

## 1 Introduction

Although there is an abundance of literature about mercury poisoning,^[1–4]^ cases involving Chinese folk prescription (CFP) have seldom been mentioned, and there are discrepancies between CFP and traditional Chinese medicine (TCM), which have yet to be articulated. It is also of note that following CFP by an unlicensed practitioner engaged in quackery (UPEQ) for TCM is one of the causes of mercury intoxication in rural areas in China. Ignorant of the deception, people could be caught unaware of their intoxication, resulting in delayed presentation to hospital. Through the case retrospectively from medical records, we address discrepancies between CFP and TCM, and the potential menace of UPEQs. By raising awareness of these dangerous practices, we can enable patients to avoid temptation of quackery in the future and remind practitioners that a high index of suspicion is needed for diagnosis and treatment.

Informed consent of the patient was obtained and observation and discussion were presented below.

## 2 Case report

A 35-year-old rural woman was admitted to Chongqing Prevention and Treatment Center for Occupational Diseases (CPOD) due to a urine mercury level of 0.71 mg/L (normal: <0.01 mg/L), from a sample she gave a week before.

The patient described how her problems started 50 days ago, when she presented to a community hospital with itchy skin. She was diagnosed with eczema, but therapy did not make significant improvements, so she sought help from a local UPEQ. Followed his folk prescription, she took almost 35 g of mercurochrome (Hg_2_O) orally. About 2 minutes later, she passed dark red bloody stools, felt nauseous, and vomited dark red mucus. She drank some 5% glucose solution, but only the dark red bloody stools resolved. However, 2 days later, she experienced dizziness, palpitations, limb weakness, blurred vision, epigastric pain, loosening of her teeth, and pyrexia. Also, 5 days after mercury consumption, without any treatment, numbness of the whole body emerged. This caused her to present to the district general hospital where she was admitted. She was again diagnosed with eczema, treatments given failed and she developed anasarca. Therefore, 30 days after mercury consumption, she was transferred to another local polyclinic where mercury poisoning was suspected. Intramuscular injection of sodium dimercaptosulphonate at a dose of 0.125 g was performed in 8 days, after which, most of her symptoms improved significantly.

To make a definitive diagnosis of mercury poisoning, 45 days after mercury consumption, a urine sample was sent to CPOD and she was transferred from the polyclinic to a bigger hospital in the city, where further investigation were done. Urine tests there showed positive result of erythrocyte, KET (ketone body) and protein. Blood tests revealed elevated triiodothyronine (T3) and thyroxine (T4) levels. Electrocardiogram showed sinus tachycardia and electroencephalogram revealed minimal abnormity. Thus, she was diagnosed with hyperthyroidism and began corrective therapy. Then, 5 days after being admitted to the city hospital she was informed of her urinary mercury level of 0.71 mg/L by the doctor of CPOD and was transferred to CPOD 2 days later. Further investigations were done at CPOD on day 1 (1 day after her admission). Urine test showed a mercury level of 0.097 mg/L. Gastroscopy revealed chronic superficial gastritis and erosive gastritis. A 3-course chelation therapy with sodium dimercaptosulphonate was performed on day 2, day 10 and day 15, after which the mercury level significantly decreased from 0.315 mg/L on day 2 to 0.045 mg/L on day 15. Her electrocardiogram and kidney function tests all returned into normal on day 15, but her gastritis did not improve by discharge. Because of the improvement of her symptoms and her family situation, the doctor approved her discharge on day 20. The doctor advised her to take regular medication at home and to come back for assessment in half a year.

## 3 Discussion

In our case, the patient was constantly tormented with unresolved skin condition and sought solution from CFP, but instead an unintentional mercury intoxication was the result. The patient was prone to seek help at district or general hospital before mercury poisoning was suspected 30 days after mercury consumption. However, in China, mercury poisoning can only be definitively diagnosed at prevention and treatment centers for occupational diseases (PTD) like CPOD or Centers for Disease Control, and treatments can only be provided at PTDs. Therefore, all suspected cases must go to PTDs. Though, the patient was ultimately transferred to CPOD. If mercury poisoning had been suspected earlier, they would have spent less time and money and received relevant treatments earlier. However, mercury poisoning is an unusual clinical presentation and difficult diagnosis to make for doctors in general hospitals.

Also, we observed an increase of T3 and T4, which we suspect was relevant to mercury intoxication. A study among US adults claimed that mercury was associated with decrease in T3 and T4^[5]^; however, relationship between mercury poisoning and thyroid function remains ambiguous. We found erythrocyte, KET, and protein in urine, which suggested that kidney lesion was considerable. Inorganic mercury in mercurous form can contributed to renal damage,^[6]^ but further examinations for precise clinical diagnosis were not performed.

In this case, the patient sought help from CFP when formal treatment failed. Although in fact western medicine is now available and convenient, TCM is still commonly used by the Chinese population.^[7]^ And because of the blurred lines between TCM and CFP, people sometimes mistake CFP for TCM. But there are key differences between TCM and CFP yet to be discussed.

TCM is also called Chinese pharmacy which is a formal medical treatment largely but not exclusively involving herbs.^[8,9]^ In contrast, CFP represents the informal use of TCM and relies mostly on experience than formal teaching, making CFP a random use of TCM without knowing its exact ratio of components and mechanism. CFP is not described in medical texts, instead imparted by word of mouth by people without medical licenses, including UPEQs, who claim to hold secret ancestral formulas.^[10]^ TCM is a formal practice of medical science just like western medicine, and study of mechanism and components of TCM is in its infancy. In contrast, CFP has not been verified by modern medicine and not been approved by government. Neither its mechanism nor its components are pinpointed. Without formal medical knowledge, proportion and dose of contents in CFPs can be random.

However, the lack of scientific background and training, plus overlaps between the content and practice of TCM and CFP, understandably makes CFP a cheaper option adding to its attractiveness as an alternative. When formal solutions to their symptoms are all ineffective, patients might seek alternatives. They may turn to UPEQs with their CFP and false promises, hoping the mysterious formulae could help. UPEQs, who do not possess medical licenses, are the primary providers of CFPs,^[11]^ alongside other illegal medical practices. UPEQs often claim that they acquire medical knowledge by themselves or are students of a famous TCM expert. However, they receive no formal medical education and their understanding of medical science is minimal. Their wanton invention and use of their own CFP can be perilous. To this day, UPEQs still can be found in China, especially in some rural and semi-urban areas where the populations’ financial resources and the communities’ medical facilities lag behind. Intending to save money, people take the risk of cheaper unknown CFP offered by unregulated UPEQs, which can lead to unexpected and disastrous results.

## 4 Conclusions

Doctors must not only have a high index of suspicion for heavy metal poisoning and recognize the constellation of signs and symptoms, but they must also take a thorough and probing history asking specifically about any other medication, TCM and CFP, and any symptom that resulted. Also, without knowing the contents and mechanism of CFP, we cannot help the public make informed decision on the benefits or risks of CFP. Therefore, we suggest that the prime way to prevent people from being afflicted by CFP is to make clear the contents and mechanism of CFP, upgrade the effective to TCM, and exclude the noneffective.
